# *Mondia whitei* (Periplocaceae) prevents and *Guibourtia tessmannii* (Caesalpiniaceae) facilitates fictive ejaculation in spinal male rats

**DOI:** 10.1186/1472-6882-13-4

**Published:** 2013-01-07

**Authors:** Pierre Watcho, Patrick Brice Deeh Defo, Modeste Wankeu-Nya, Miguel Carro-Juarez, Telesphore Benoît Nguelefack, Albert Kamanyi

**Affiliations:** 1 Animal Physiology and Phytopharmacology Laboratory, University of Dschang, Box 67, Dschang, Cameroon; 2 Laboratorio de Comportamiento Reproductivo, Escuela de Medicina Veterinaria y Zootecnia, Universidad Autónoma de Tlaxcala, Tlaxcala, C.P, 90000, Mexico

**Keywords:** *Mondia whitei*, Guibourtia tessmannii, Ejaculation, Spinal cord, Rat

## Abstract

**Background:**

*Mondia whitei* and *Guibourtia tessmannii* are used in Cameroon traditional medicine as aphrodisiacs. The present study was undertaken to evaluate the pro-ejaculatory effects of the aqueous and organic solvent extracts of these plants in spinal male rats.

**Methods:**

In spinal cord transected and urethane-anesthetized rats, two electrodes where inserted into the bulbospongiosus muscles and the ejaculatory motor pattern was recorded on a polygraph after urethral and penile stimulations, intravenous injection of saline (0.1 ml/100 g), dopamine (0.1 μM/kg), aqueous and organic solvent plant extracts (20 mg/kg).

**Results:**

In all spinal rats, urethral and penile stimulations always induced the ejaculatory motor pattern. Aqueous or hexane extract of *Mondia whitei* (20 mg/kg) prevented the expression of the ejaculatory motor pattern. The pro-ejaculatory effects of dopamine (0.1 μM/kg) were not abolished in spinal rats pre-treated with *Mondia whitei* extracts. Aqueous and methanolic stem bark extracts of *Guibourtia tessmannii* (20 mg/kg) induced fictive ejaculation characterized by rhythmic contractions of the bulbospongiosus muscles followed sometimes with expulsion of seminal plugs. In rats pre-treated with haloperidol (0.26 μM/kg), no ejaculatory motor pattern was recorded after intravenous injection of *Guibourtia tessmannii* extracts (20 mg/kg).

**Conclusion:**

These results show that *Mondia whitei* possesses preventive effects on the expression of fictive ejaculation in spinal male rats, which is not mediated through dopaminergic pathway; on the contrary, the pro-ejaculatory activities of *Guibourtia tessmannii* require the integrity of dopaminergic system to exert its effects. The present findings further justify the ethno-medicinal claims of Mondia whitei and *Guibourtia tessmannii*.

## Background

Ejaculation is the physiological process that describes the expulsion of the semen from the urethra and comprises two successive phases, emission and expulsion and requires the participation of different pelvi-perineal anatomical structures [1-3]. The emission phase consists of secretion of the various components of semen by seminal vesicles, prostate and through release of ampullary vasa deferentia content into the prostatic urethra. The expulsion phase corresponds to forceful propulsion of sperm from the prostatic urethra to the urethral meatus through rhythmic contractions of perineal striated muscles, with a primary role for the bulbospongiosus muscle (BS) [4]. Ejaculation is controlled by the central nervous system both at brain and spinal levels.

In the brain, experimental evidence suggests the existence of a cerebral ejaculation-related subcircuit within a larger neural circuitry involved in male sexual behavior [5]. At the spinal level, evidence has shown the existence of a generator that controls both phases of ejaculation, located within the lumbosacral spinal cord [6,7]. This ejaculation generator is under the influence of supraspinal sites of the brain stem and hypothalamus [6,8].

A growing number of international studies on sexual health issues suggest that many men worldwide have sexual health problems mainly centered in the ejaculatory function [9,10]. Particularly, premature or rapid ejaculation is the most common male ejaculatory dysfunctions and its prevalence is estimated at approximately 30% across all age groups [11]. Many pharmaceutical drugs like fluoxetin, paroxetin, haloperidol and serotonin have been exploited to control ejaculatory response [12,13]; but these compounds have significant side effects [13,14]. Several studies have shown that medicinal plants with aphrodisiac properties are viable alternatives with anecdotal evidence for their effectiveness in the management of sexual disorders including ejaculation dysfunctions [15-17]. Aphrodisiacs are substances able to excite libido or arouse sexual instinct and can be categorized according to their mode of action into three groups: by increasing libido (i.e. sexual desire), by increasing potency (i.e. effectiveness of erection) and by increasing sexual pleasure [18]. These substances act at the CNS system by altering specific neurotransmitters or sex hormone concentrations [16-21]. Aphrodisiacs mainly derived from plant species act on specific neurotransmitter systems. For instance, previous results from our research group have shown that an intravenous injection of extracts from *Bersama engleriana*[21] and *Dracaena arborea* (unpublished) to spinal male rats prevent the rhythmic contractions of the bulbospongiosus muscles (the main ejaculatory muscles) and the occurrence of ejaculation induced by dopamine and oxytocin. *Mondia whitei* (Periplocaceae) and *Guibourtia tessmannii* (Caesalpiniaceae) used in the present study also belong to this large group of aphrodisiac plants.

*Mondia whitei* is a woody climber with a large tuberous root stock; it is widely distributed in tropical Africa, from Guinea through Cameroon to East Africa. In Cameroon, *Mondia whitei* is referred to as "Limte", "Nkang bongo", "Yang" or "la racine". The roots are used either as spices, aphrodisiacs or for the treatment of urinary tract infection, jaundice and headache, while the whole plant is used to treat diarrhea [22,23]. By testing various extracts from the roots of *Mondia whitei*, we reported that the aqueous and hexane extracts induced an androgenic effect [24,25], promoted vas deferens and corpus cavernosum relaxation [26] and facilitated the expression of sexual behaviour [27].

Traditional medicine reports that *Guibourtia tessmannii* also referred to as ``Essingang`` or ``Bubinga`` is a tall size tree (40-50 m) widespread throughout tropical Africa and America and, preferring higher rainfall or evergreen forests. This plant is distributed from Cameroon to Democratic Republic of Congo and parts of southern America. In the Centre Region of Cameroon, the stem barks are used for the treatment of cardiovascular diseases [28], cancer [29] and as aphrodisiacs. To the best of our knowledge, no work has been carried out to scientifically justify the effects of *Guibourtia tessmannii* on sexual behavior. With the hypothesis that the bioactive compounds present in *Mondia whitei* and *Guibourtia tessmannii* possess an aphrodisiac effect acting upon the ejaculatory circuitry, the present study was undertaken to evaluate the ejaculatory properties of aqueous and organic extracts of *Mondia whitei and Guibourtia tessmannii* in spinal cord transected and urethane-anesthetized rats. We employed the fictive ejaculation model in spinally and urethane-anaesthetized male rats [21,30]. This model permits the recording and visualization of the rhythmic motor pattern of ejaculation accompanied by complex pelvic activity that includes phasic and strong penile erections, as well as penile movements followed by the potent expulsion of urethral contents. The rhythmic motor pattern of ejaculation registered in this experimental model is elicited by urethral and penile stimulations and can additionally be induced by systemic administration of several drugs including medicinal plant extracts [3,19-21]. Growing evidence shows that dopamine is a neurotransmitter playing a central role in the control of ejaculation [31,32]. Thus, for instance, stimulation of dopaminergic receptors by systemic dopamine [21] or apomorphine [33,34] provokes the expression of ejaculation in spinal male rats. On the other side, systemic administration of antagonists of dopaminergic receptors abolishes the expression of the ejaculatory response in spinal rats [35,36]. Accordingly, in the present study, the effects of the plant extracts were also investigated in the presence or absence of dopamine or its nonspecific antagonist, haloperidol.

## Methods

### Collection of plant material and preparation of extracts Mondia whitei collection

Fresh roots of *Mondia whitei* were collected in December 2010 in Bangangté, Nde Division, West Region, Cameroon. Botanical identification was done at the Cameroon National Herbarium (HNC) in Yaoundé in comparison with the Herbarium Voucher specimen No42920/HNC. The roots of *Mondia whitei* were cut into small pieces of about 1.5-2 cm, shade-dried and powdered using an electric grinder (Moulinex).

### Mondia whitei extracts Aqueous extract

A total of 250 g of the powder of *Mondia whitei* roots were macerated in 1.5 L of distilled water for 72 hours. The macerate was filtered and the filtrate was oven dried (55°C). The resulting material was found to weight 23 g (9.2% yield, w/w based on the dried starting weight). The working solution was obtained by dissolving 1 g of the residue in a known volume of saline solution and the final volume adjusted to 10 ml with the same solvent.

### Hexane extract

A total of 300 g of the powder of *Mondia whitei* roots were macerated in 1 L of hexane for 72 hours to yield, after solvent evaporation under reduced pressure, 1.145 g of brownish extract corresponding to an extraction yield of 0.38% (w/w based on the dried starting weight). The working hexane solution was obtained by dissolving 1 g of the residue in 1 ml of tween 20 and the final volume adjusted to 10 ml with saline solution.

### Guibourtia tessmannii collection

Fresh stem barks of *Guibourtia tessmannii* were collected in December 2010 in Ngoumou, Mefou-Akono Division, Center Region, Cameroon. Botanical identification was performed in the Cameroon National Herbarium (HNC) in comparison with the existing Herbarium Voucher specimen 1037/SRFCA. The stem bark was shade-dried and powdered using an electric grinder (Moulinex).

### Guibourtia tessmannii extracts Aqueous extract

A total of 250 g of the powder of *Guibourtia tessmannii* were macerated in 1.5 L of distilled water for 72 hours. The macerate was filtered and the filtrate was oven dried (55°C). The resulting material was found to weight 37.6 g (15.04% yield, w/w based on the dried starting weight). The working solution was obtained by dissolving 1 g of the residue in a known volume of saline solution and the final volume adjusted to 10 ml with the same solvent.

### Methanolic extract

A total of 300 g of the powder of *Guibourtia tessmannii* were macerated in 1.5 L of methanol for 72 hours to yield, after solvent evaporation under reduced pressure, 34.1 g of brownish extract corresponding to an extraction yield of 11.37% (w/w based on the dried starting weight).

### Phytochemical screening

The aqueous and organic extracts of *Mondia whitei* and *Guibourtia tessmannii* were screened for the presence or absence of groups of triterpenes, reducing sugars, phenolics and phytosterols using the following standard techniques [37-40]:

### Iron chloride test

This test detects the presence of phenyl compounds. In a test tube, some drops of the plant extract are added to methanol and then a few drops of methanol added. The presence of phenyl compounds is marked by the appearance of a blue or violet coloration.

### Liebermann-Burchard test

This test detects the presence of steroid and triterpenes. In a test tube, some drops of the plant extract are added to chloroform followed by a few drops of acetic anhydride and concentrated sulfuric acid. The presence of steroids is marked by the appearance of a greenish blue coloration while triterpenes are revealed by a red violet coloration.

### Molish test

This test detects the presence of sugars. In a test tube, some drops of the plant extract are added to ethanol and ethanolic alpha naphtol. After homogenization, a few drops of concentrated sulfuric acid are equally added. The presence of sugars is marked by the presence of a red violet rind at the interphase.

### Flavonoid test

This test detects the presence of flavonoids. In a test tube, some drops of the plant extract are dissolved in methanol followed by addition of a few drops of concentrated hydrochloric acid and powdered magnesium. The presence of flavonoids is marked by the appearance of a pink, orange or purple coloration and triterpenes by a red violet coloration.

#### Animals

Healthy and sexually experienced adult male Wistar rats (> 90 days, 200–300 g body weight) were obtained from the animal house of the Department of Animal Biology of the University of Dschang. They were housed in groups (four rats per cage), under natural LD cycle and with free access to food and water. The experiments were performed in accordance with the internationally accepted standard ethical guidelines for laboratory animal use and care as described in the European Community guidelines; EEC Directive 86/609/EEC, of the 24th November 1986 [41].

#### Sexual training

To provide sexual experience, each male rat was submitted to five consecutive sexual behavioural tests with an ovariectomized female brought to heat (estrus) artificially with a single subcutaneous dose of 30 μg estradiol benzoate and 600 μg progesterone, 48 h and 6 h before testing. In ovariectomized rat, it was shown that estradiol benzoate induced a specific urge to seek contact with a sexual active male [42]. Behavioural observations were conducted after the onset of darkness and males were individually introduced into a cylindrical observation cage and an adaptation period (5-10 min) was allowed. Then, a receptive stimulus female was introduced and the copulatory behaviour was permitted during a period of 25 min. Male rats exhibiting active sexual behaviour and ejaculation latencies of less than 15 min in the last three sessions were used to classify them as sexually experienced male rats.

#### Drugs

Dopamine, urethane, estradiol, progesterone (Sigma Chemicals, St Louis, USA) and haloperidol (MP Biomedicals, Inc, Germany) were used during this study. Dopamine, urethane and haloperidol were freshly prepared in saline solution. Estradiol and progesterone were dissolved in ethanol and administered in oil. Doses of dopamine (0.1 μM/kg) and haloperidol (0.26 μM/kg) were selected based on previous studies (21,26). For each substance, the volume administered intravenously was 0.2 ml/rat while the infusion time was 5 s.

#### Surgical preparation

Animals were urethane-anesthetized (1.5 g/kg intraperitoneally), and by performing a surgical incision on the perineum, the bulbospongiosus genital muscles were identified and exposed. Two electrodes (EL 452, 12 mm, BIOPAC) were inserted into the bulbospongiosus muscles to record electromyographic (EMG) activity. For a better visualization of the motor genital activity associated with the ejaculation, an additional surgery was performed to expose the bulbar portion of the penis and its anatomical connections with the striated bulbospongiosus muscles. At the end of the surgical approach, the spinal cord was blunt transected around T6 spinal level and prepared for recording [19]. Treatments were administered by infusing the selected extracts and compounds into the jugular vein.

#### Experimental treatment

Animals were randomly divided into 12 groups of five rats each and intravenously treated as follows: Group 1, saline solution or tween in saline solution (1 ml/kg, control); Group 2, aqueous extract of *Mondia whitei* (20 mg/kg); Group 3, hexane extract of *Mondia whitei* (20 mg/kg); Group 4, aqueous extract of *Guibourtia tessmannii* (20 mg/kg); Group 5, methanolic extract of *Guibourtia tessmannii* (20 mg/kg); Group 6, dopamine (0.1 μM/kg); Group 7, aqueous extract of *Mondia whitei* (20 mg/kg) plus dopamine (0.1 μM/kg); Group 8, hexane extract of *Mondia whitei* (20 mg/kg) plus dopamine (0.1 μM/kg); Group 9, haloperidol (0.26 μM/kg); group 10, haloperidol (0.26 μM/kg) plus methanolic extract of *Guibourtia tessmannii* (20 mg/kg). In all sequential treatments (Groups 7, 8, 10), the second drug application was performed 3 minutes after the first one. The doses of plant extracts were chosen on the basis of our pilot studies (unpublished) and the infusion time was 5 s.

#### Activation and recording of the rhythmic genital motor pattern of ejaculation

Immediately after spinal cord transection, ejaculatory motor patterns could be reflexively expressed and recorded in the genital muscles of all animals. To establish the capacity of the spinal apparatus to produce the genital rhythmic pattern after spinalization, two consecutive ejaculatory motor patterns were repeatedly evoked at 3-min intervals by the injection of saline solution (200 μl/min) through a PE-50 catheter (0.965 mm o.d.) inserted into the pelvic urethra through a bladder incision. Injection of saline solution was directed to increase the intraurethral pressure to simulate the urethral distention produced by the emptying of the contents of the accessory glands into the posterior urethra. Thereafter, one of the selected treatments was intravenously applied and the number, frequency of contractions of the striated bulbospongiosus muscles and its latency of response obtained under their influence were recorded for 5 min, which was registered on a polygraph (Biopac Student Lab PRO, version 3.7.3, frequency 50Hz and model MP36E-CE).

Five minutes after recording the EMG in each sequential treatment, three consecutive urethral stimulations were monitored at 3 min intervals, as described above. The latency of contractions was expressed as the time elapsed from the application of a test stimulus until the first contraction of the bulbospongiosus muscles. The number of motor contractions included all motor contractions expressed in the motor ejaculatory train evoked by the sensorial or pharmacological stimuli. The frequency of contractions of the bulbospongiosus muscles was calculated by dividing the number of contractions by the duration of the motor train.

#### Statistical analysis

Data are reported as the mean plus standard error of mean (SEM). Significance was calculated by one-way analysis of variance (ANOVA) followed with post-hoc Tukey HSD test for multiple comparisons. p values < 0.05 were considered significant. The statistical tests were performed with Stat Soft, Inc. (2008). STATISTICA (data analyses software system), version 8.0. http://www.statsoft.com.

## Results

### Phytochemical screening

Phytochemical screening of the aqueous and hexane extracts of *Mondia whitei* showed reducing sugars, triterpenes and steroids while phenolic compounds were found in *Guibourtia tessmannii* extracts (Table 1).

**Table 1:** **Results of the phytochemical screening of *Mondia whitei and Guibourtia tessmannii *extracts**

**Group of compounds**	***Mondia whitei***		***Guibourtia tessmannii***	
	**Aqueous extract**	**Hexane extract**	**Aqueous extract**	**Methanolic extract**
Reducing sugars	+	+	-	-
Triterpenes	+	+	-	-
Steroids	+	+	-	-
Phenolic compounds	-	-	+	+
Flavonoid	-	-	-	-

**+**: present - : absent.

### Activation of the ejaculatory motor response by urethral and penile stimulations

**Figure 1:**
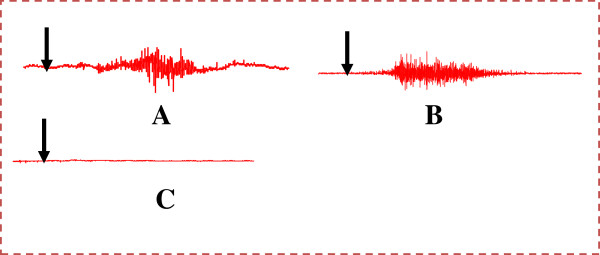
**Polygraphic EMG tracings showing the effects of mechanical and pharmacological stimulations upon the expression of the fictive ejaculation response in spinal rats: (A)Urethral stimulation; (B)Penile stimulation; (C)Intravenous injection of saline solution.** Arrows indicate the moment of injection.

In all spinal cord transected and urethane-anesthetized rats, injection of saline solution (200 μl/min) into the pelvic urethra (urethral stimulation) before each drug administration provoked rapid rhythmic contractions of the striated bulbospongiosus muscles with an average mean of 4.57 ± 0.81 contractions (Figure 1A and Table 2). In some cases, an expulsion of the urethral contents accompanied the ejaculatory rhythmic contractions and was always accompanied by penile movements and penile erections. Penile stimulation also provoked the rhythmic ejaculatory pattern with an average mean of 8.52 ± 1.59 contractions (Figure 1B and Table 2). Penile stimulation appeared to be more efficient than urethral stimulation to provoke rapid response (latency of contractions: 2.53 ± 0.8 s). Intravenous administration of saline solution or tween in saline solution (not shown) did not provoke fictive ejaculation (Figure 1C, and Table 2).

**Table 2:** Effects of urethral and penile stimulations, intravenous administration of drugs on the number, frequency and latency of contractions of the bulbospongiosus muscles in spinal rats

**Treatment**	**Number of contractions (N)**	**Frequency of contractions (*N*** **s-**^**1**^**)**	**Latency of contractions (s)**
Urethral stimulation	4.57 ± 0.81	0.45 ± 0.03	28.11 ± 5.65
Penile stimulation	8.52 ± 1.59	0.34 ± 0.05	2.53 ± 0.80
Dopamine (0.1 μM/kg)	9.80 ± 0.86^###^	0.41 ± 0.02	11.89 ± 2.35^###,ФФФ^
Aqueous extract of *Guibourtia tessmannii* 20 mg/kg	4.80 ± 1.15^Ф,***^	0.38 ± 0.05	284.02 ± 75.10^###,ФФФ,***^
Methanolic extract of *Guibourtia tessmannii* 20 mg/kg	3.80 ± 0.93^ФФ,***^	0.38 ± 0.04	150.86 ± 36.98^###,ФФФ,***^
Aqueous extract of *Mondia whitei* (20 mg/kg) plus dopamine (0.1 μM/kg)	6.66 ± 0.66	0.48 ± 0.06	ND
Hexane extract of *Mondia whitei* (20 mg/kg) plus dopamine (0.1 μM/kg)	6.67 ± 0.89	0.46 ± 0.02	ND

Number of rats per group = 5. All values are expressed as mean ± SEM. Urethral and penile stimulations represent the mean value of all urethral and penile stimulations carried out in this study (three stimulations per rat).

^###^ p < 0.001 significantly different compared with urethral stimulation.

^Ф^ p < 0.05, ^ФФ^ p < 0.01 and ^ФФФ^ p < 0.001significantly different compared with penile stimulation.

^*^p < 0.05, ^**^p < 0.01 and ^***^ p < 0.001 significantly different compared with dopamine.

For each rat, the frequency of contractions of the bulbospongiosus muscles was calculated by dividing the number of contractions (N) by the duration of the motor train and the latency was observed during EMG recording. ND = not determined.

### Activation of the ejaculatory motor response by dopamine and haloperidol administration

**Figure 2:**
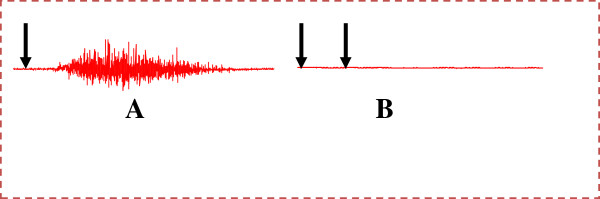
**Polygraphic EMG tracings showing the effects of mechanical and pharmacological stimulations upon the expression of the fictive ejaculation response in spinal rats: (A)Intravenous injection of dopamine (0.1 μM/kg); (B)Intravenous injection of haloperidol (0.26 μM/kg) (first arrow) followed by the injection of dopamine (0.1 μM/kg) (second arrow).** Arrows indicate the moment of injection.

Dopamine (0.1 μM/kg) administration provoked the rhythmic ejaculatory pattern with an average mean of 9.80 ± 0.86 contractions (Figure 2A, and Table 2) corresponding to an ejaculatory latency of 11.89 ± 2.35 s. Intravenous administration of haloperidol (0.26 μM/kg), a nonspecific antagonist dopamine receptor, resulted in no contraction of the bulbospongiosus muscles (Figure 2B, and Table 3).

**Table 3:** Effects of intravenous administration of drugs on the occurrence of ejaculatory motor response

**Treatment**	**Ejaculatory motor response**
Saline solution (0.9%) (0.1 ml/kg)	-
Dopamine (0.1 μM/kg)	+
Haloperidol (0.26 μM/kg)	-
Aqueous extract of *Mondia whitei,* 20 mg/kg	-
Aqueous extract of *Mondia whitei* (20 mg/kg) plus dopamine (0.1 μM/kg)	+
Hexane extract of *Mondia whitei,* 20 mg/kg	-
Hexane extract of *Mondia whitei* (20 mg/kg) plus dopamine (0.1 μM/kg)	+
Aqueous extract of *Guibourtia tessmannii,* 20 mg/kg	+
Methanolic extract of *Guibourtia tessmannii,* 20 mg/kg	+
Haloperidol (0.26 μM/kg) plus methanolic extract of *Guibourtia tessmannii* (20 mg/kg)	-

Number of rats per group = 5. + = present - = absent.

### Effects of Mondia whitei and Guibourtia tessmannii extracts on fictive ejaculation

**Figure 3:**
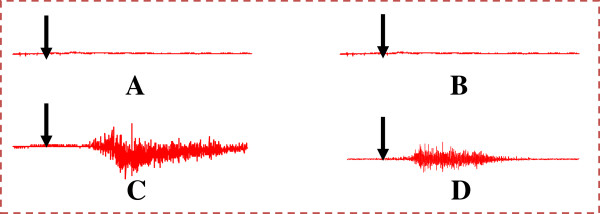
**Polygraphic EMG tracings showing the effects of plant extracts: (A)Aqueous extract of *Mondia whitei* (20 mg/kg); (B)Hexane extract of *Mondia whitei* (20 mg/kg); (C)Aqueous extract of *Guibourtia tessmannii* (20 mg/kg); (D)Methanolic extract of *Guibourtia tessmannii* (20 mg/kg).** Arrows indicate the moment of injection.

Intravenous administration of either the aqueous extract (20 mg/kg) or the hexane extract (20 mg/kg) of *Mondia whitei* in spinal cord transected and urethane-anesthetized rats did not provoke the activation of the ejaculatory motor pattern. These effects were similar to those obtained after intravenous saline injection (0.1 ml per 100 g of bw) (Figure 3A, B, and Table 3) or tween in saline solution (not shown). On the other hand, the aqueous and methanolic extracts of *Guibourtia tessmannii* (20 mg/kg) induced fictive ejaculation, characterized by rhythmic contraction of the bulbospongiosus muscles and followed sometimes with expulsion of seminal plugs (Figure 3C, D, and Table 2). These *Guibourtia tessmannii* induced-contractions appeared with a significant delay when compared to dopamine (p < 0.001).

### Effects of the aqueous and hexane extracts of Mondia whitei on the expression of dopamine-induced fictive ejaculation

**Figure 4:**
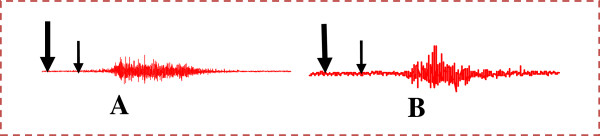
**Polygraphic EMG tracings showing the effects of sequential treatment of *Mondia whitei *plus dopamine; (A)Aqueous extract of *Mondia whitei* (20 mg/kg) plus dopamine (0.1 μM/kg); (B)Hexane extract of *Mondia whitei* (20 mg/kg) plus dopamine (0.1 μM/kg).** Long and thick arrows indicate plant extract injection while short and thin arrows show dopamine injection.

The evidence that dopamine (0.1 μM/kg) induced fictive ejaculation was shown in Figure 2A and Table 2. This pro-ejaculatory effect of dopamine was not prevented in rats pre-treated with *Mondia whitei* extracts (20 mg/kg) (Figure 4A, B, and Table 3).

### Effects of haloperidol on the expression of Guibourtia tessmannii-induced fictive ejaculation

**Figure 5:**
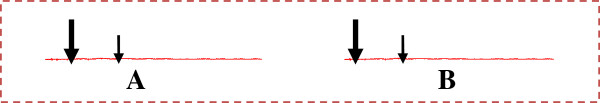
**Polygraphic EMG tracings showing the inhibiting effects of haloperidol on the aqueous (A) and methanolic (B) extracts of *Guibourtia tessmannii-*induced fictive ejaculation.** Long and thick arrows indicate haloperidol injection while short and thin arrows show *Guibourtia tessmannii* injection.

Pre-treated rats with haloperidol, a non-specific antagonist of dopamine receptors, prevented the *Guirboutia tessmannii* methanolic extract-induced expression of EMG activity of the bulbospongiosus muscles in all rats (Figure 5A, B, and Table 3).

## Discussion

In the present study, we observed that the fictive ejaculation response was prevented after the intravenous administration of the aqueous and hexane extracts of *Mondia whitei* (20 mg/kg) and facilitated after systemic injection of *Guibourtia tessmannii* (20 mg/kg) extracts.

Data obtained with *Mondia whitei* show that the bioactive compounds present in this medicinal plant exert a delaying effect at the level of the spinal cord to block the expression of the ejaculatory motor pattern. However, when compared to the androgenic and pro-sexual potentials of *Mondia whitei* shown in male rats [24,27], it could clearly be proposed that the crude extracts from this plant act at different levels to promote male sexual potency and fertility. In agreement with Sandroni [18], substances that increase sexual potency are considered as aphrodisiac compounds [27,43] and its influence is directly observed on male sexual reflexes, including ejaculation. Present findings in spinal male rats show that the aphrodisiac effects of *Mondia whitei* previously reported in copulating male rats [27] may not be promoted by an increase in sexual potency. Thus, it seems that the compounds contained in *Mondia whitei* extracts act differentially on various structures of the central nervous system involved in the expression of ejaculation by exerting a facilitatory pro-sexual effect on brain structures or by exerting inhibitory effects on spinal circuits. In an attempt to determine the possible targets and mechanism(s) of action of *Mondia whitei* in the delay of ejaculation observed in the present work, the ejaculation-preventing effects of *Mondia whitei* were evaluated on dopamine-induced ejaculation in spinalized animals. Thus, the pro-ejaculatory effect of dopamine (0.1 μM/kg) was not abolished in spinal rats pre-treated with *Mondia whitei* extracts. It could therefore be thought that *Mondia whitei* possesses preventive effects on the expression of fictive ejaculation at the spinal level which are not mediated through dopaminergic pathway. In copulating male rats, it has been shown that *Mondia whitei* exerts its aphrodisiac effects by acting upon the dopaminergic system but the precise site of action is not known at present [27]. Present data suggest that the preventing actions of *Mondia whitei* on the expression of ejaculatory response are not exerted at the level of the spinal circuits that structure the ejaculation generator but instead, a main role for higher nervous structures in the mediation of its aphrodisiac actions can be suggested. Present finding further suggests that *Mondia whitei* could be a good candidate for patients with rapid ejaculation in order to prevent the prompt expression of ejaculation.

It was observed in the present work that *Guibourtia tessmannii* extracts can facilitate ejaculatory response in male rats and therefore give a first account for the claimed pro-sexual properties of this plant. The aqueous and methanolic stem bark extracts of *Guibourtia tessmannii-*induced fictive ejaculation was characterized by rhythmic contraction of the bulbospongiosus muscles, followed sometimes with the expulsion of seminal plugs and by a significant increase in its latency of expression. This significantly long latency promoted by *Guibourtia tessmannii* extracts suggests that the aphrodisiac potentials of the plant may be related to the promotion of sexual potency [18] i.e. the promotion of the ejaculatory motor trains. As occurred with other previously reported aphrodisiac plant extracts [19,20], it seems that the compounds contained in the aqueous and methanolic extracts of *Guibourtia tessmannii* exert their actions by targeting the spinal circuits in charge of ejaculation control.

Besides, a pre-treatment of rats with haloperidol (0.26 μM/kg), a non-specific antagonist for all dopamine receptors, completely abolished the aphrodisiac effect of *Guibourtia tessmannii.* These results denote the participation of the spinal dopaminergic pathways. In line with the present results, it has been found that some plant extracts with aphrodisiac actions such as *Bersama engleriana*[21] prolong the ejaculatory latency and the occurrence of fictive ejaculation induced by dopamine through the blockade of dopaminergic receptors in rats. Thus, it appears that the dopaminergic system plays a main potential role in the mediation of the pro-sexual actions evoked by aphrodisiac plants and further supports the notion that the presumed dopaminergic receptors that are targeted by *Guibourtia tessmannii* are located on the neurons of the ejaculation generator. Specific studies are necessary to test these possibilities.

Fictive ejaculation is a physiological response registered in urethane-anaesthetized and spinal rats characterized by the rhythmic contractions of bulbospongiosus muscles [30]. It has been demonstrated that fictive ejaculation can be pharmacologically induced by the systemic injection of dopamine or apomorphine acting at the spinal generator for ejaculation [21,33,34,44] and it has been considered as the main model for the study of the spinal pattern generator of ejaculation [8]. Thus, with no doubt, the effect of the aphrodisiac plants could be systemically evaluated by using the fictive ejaculation model and particular results, highlight the differential effects of aphrodisiac plants.

## Conclusion

Present results show that *Mondia whitei* extracts could be considered as an ejaculation blocking agent that prevents the expression of fictive ejaculation, an effect that is not mediated through the dopaminergic pathway. On the other hand, *Guibourtia tessmannii*-induced fictive ejaculation requires principally the integrity of the dopaminergic system. Further investigation of the effects of these two medicinal plants in animals with real ejaculatory dysfunction such as diabetic or obese rats is highly needed.

## Competing interests

We declare that we have no competing of interests.

## Authors’ contributions

PW conceived the project and wrote the final draft of the protocol. PW, PBDD, MCJ and TBN did the literature search and assist in the methodology. PW and PBDD contributed to the laboratory work, data analysis and data interpretations. MCJ helped in the writing and reviewing process. AK was responsible for the overall supervision. PW, PBDD and MWN wrote the paper with input from all the authors. All authors read and approved the final manuscript.

## Pre-publication history

The pre-publication history for this paper can be accessed here:

http://www.biomedcentral.com/1472-6882/13/4/prepub
